# Organizational health literacy in German hospitals: a cross-sectional survey among hospital managers

**DOI:** 10.1186/s12913-024-11649-x

**Published:** 2024-10-13

**Authors:** Nicola Häberle, Jonas Lander, Marie-Luise Dierks, Eva Maria Bitzer

**Affiliations:** 1 Department of Public Health & Health Education, University of Education Freiburg, Kunzenweg 21, 79117 Freiburg, Germany; 2 Institute of Epidemiology, Social Medicine and Health Systems Research/ OE 5410, Hannover Medical School, Carl-Neuberg-Str. 1, 30625 Hannover, OE Germany

**Keywords:** Organizational health literacy, Health literacy environment, Hospital, Hospital management, Health care, Patient participation

## Abstract

**Background:**

Organizational health literacy (OHL) describes conditions and measures in healthcare institutions to enable patients to make good health-related decisions. By providing easy access to and appropriate communication of understandable information to use and navigate the facility, healthcare organizations can contribute to strengthening patients’ health literacy and self-management. The extent of OHL implementation in German hospitals remains largely unknown. This study aims to fill this gap in our knowledge by investigating OHL-related activities reported by hospital managers.

**Methods:**

Between November and December 2022, we conducted a national online survey among medical, nursing and administrative hospital managers with hospitals that operate more than 50 beds. The data were collected via the health literate health care organization ten item questionnaire (HLHO-10) and supplemented by sociodemographic questions and an open-ended question. We applied variance and correlation analyses to investigate the data.

**Results:**

Of 3,301 invited hospital managers, 371 participated in the survey (response rate 11%). The overall mean score for HLHO-10 was 4.6 (SD = 1.1) on a 7-point Likert scale, indicating a moderate level of OHL implementation. Hospital managers stated that hospitals concentrate on helping patients find their way around and communicating the costs of treatment transparently and clearly; conversely, that active patient participation in the design and evaluation of health information is rare in care settings, and that health information is seldom provided to patients through a range of media. For the practical implementation of the OHL, most hospital managers mentioned activities regarding communication standards, such as providing information materials.

**Conclusions:**

Given their unique position as hubs of human interaction, hospitals provide an ideal opportunity to promote the adoption of OHL. By actively involving patients, hospitals can better tailor their approaches to meet patient needs and preferences. Compared to studies from oncology centres in Germany and 20 Italian hospitals, the average HLHO-10 score of this study is lower. While some aspects of OHL are already embedded in inpatient care, it is imperative that OHL is thoroughly embedded in the hospitals’ organizational culture and plays a fundamental role in the daily operations of the institution. This could be done, for instance, by more explicitly addressing the topic of health literacy in staff communication training.

**Supplementary Information:**

The online version contains supplementary material available at 10.1186/s12913-024-11649-x.

## Background

Current definitions and concepts of health literacy (HL) propose that successful access to, understanding and appraisal of, and application of health information results from an individual’s abilities and characteristics interacting with societal structures and organizational conditions [1–3]. Countries such as Germany have undertaken comprehensive initiatives to promote HL across all levels of the health system and to establish the necessary standards for its development and implementation [4], such as the National Action Plan on Health Literacy [5] and the Alliance for Health Literacy [6]. However, HL still remains poor for considerable parts of society – in Germany, recent representative findings show that 59% of the population report difficulties in handling health information appropriately – with particular difficulties among people of low education or social status, and older people [7]. Meanwhile, there is evidence that an increased level of HL of patients is associated with higher level of empowerment, better decision-making skills, and a more active role in treatment [8, 9].

Health care organizations and institutions are increasingly seen having a responsibility to respond not just to the general health literacy needs of individuals [10], but also to the specific needs of vulnerable groups [11]; thus, to exhibit organizational health literacy (OHL). The term OHL reached widespread attention after Brach and colleagues published the ten attributes of OHL such as “Has leadership that makes health literacy integral to its mission, structure, and operations”, “Prepares the workforce to be health literate and monitors progress”, and “Provides easy access to health information and services and navigation assistance” [12, 13] (see Table 2 for details of the ten attributes). Two further frameworks have built on the initial concept. First, the nine standards of the “Vienna Concept of Health-Literate Hospitals and Healthcare Organizations” (V-HLO) [14, 15] connects OHL explicitly and closely to quality management, and focuses on the health literacy of patients, organizations, healthcare providers and populations. On the basis of feedback from different national contexts, V-HLO has been revised to eight standards called the “International Self-Assessment Tool for Organizational Health Literacy (Responsiveness) of Hospitals” (OHL-Hos) [14]. The second framework, from Australia, is the “Organizational Health Literacy Responsiveness framework” (Org-HLR), which has six dimensions. The self-assessment of health literacy is included in a comprehensive assessment process and is part of a trilogy of organizational development steps with corresponding tools: reflection, self-assessment, and prioritization [15–18].

Overall, implementing OHL principles might increase efficiency, eliminate redundancy and promote patient engagement, understanding and support at different stages such as prevention, decision-making and self-management, thereby supporting patient-centred care [12, 19–23]. These positive effects could also be beneficial for health expenditure [24].

So far, health professionals have implemented activities to improve OHL in healthcare organizations under the umbrella of quality management and health promotion, but have rarely labelled these activities as OHL-related [25–28]. Health professionals are seen as HL-promoters given their central position in the organizations [29–31]. To continuously embed OHL into care organisations, leadership plays a substantive role [25, 32, 33].

There are more than 20 instruments to measure OHL, mainly designed for self-assessment of health care institutions, and they vary considerably with regard to length and comprehensiveness [34]. A brief instrument, that is suitable not only for self-assessment but also for larger quantitative surveys, is the health literate health care organization ten item questionnaire (HLHO-10), which is based on the ten attributes of health-literate health care organizations [35]. The results of its validation study in 51 German breast care centres indicate satisfactory psychometric properties [35, 36]. Comparing the ten attributes among breast centres, the highest scores were in the facilities that ensured patients have truly understood everything, particularly in critical situations (e.g. medication, surgical consent). Overall, their performance was lacking, as they provided little personalized health information (e.g. health information in different languages, or in Braille) [35]. Due to its brevity, theoretical foundation and psychometric properties, we chose HLHO-10 to assess the degree to which hospitals in Germany help patients to navigate, understand, and use information and services [35].

Our study aims to assess the level of implementation of the ten attributes of OHL in German hospitals as perceived by hospital managers. By including different types and sizes of hospitals we aim to provide a comprehensive overview of the organizational health literacy in acute care hospitals, and highlight key elements unique to each hospital characteristic. We target the survey at senior hospital management: medical directors, directors of nursing and/or administrative directors. We hypothesized that targeting up to three management positions per hospital would increase the likelihood of receiving at least one response per hospital, ideally from the director responsible for OHL.

## Methods

### Study design and participants

This cross-sectional semi-standardized online and postal survey was conducted among the senior management of German acute care hospitals with more than 50 beds. We included all such hospitals listed in the German hospital report according to § 108 Social Code Book V (Location directory: https://www.dkgev.de/themen/digitalisierung-daten/informationstechnik-im-krankenhaus/verzeichnisse-und-register/), regardless of their legal entity (public, charitable, for-profit) (*n* = 1,476). Not included were day-care hospitals, specialist clinics, and rehabilitation clinics. Senior management addressed medical directors, directors of nursing, and/or administrative directors. Their contact details were manually extracted from the websites of the respective hospitals, and were not available for approximately 15% (*n* = 226) of the included hospitals. The final sample to be invited to the main survey comprised 3,301 hospital directors (medical, nursing administration) from 1,250 hospitals.

### Recruitment

We ran the survey between November 24 and December 15, 2022. We mailed the invitation to participate to up to the three hospital directors or their designated proxy or their secretary, and asked to complete the survey either digitally or per paper.

The survey was designed according to the Dillman’s method—we sent two reminders to return the questionnaire [37]. As an incentive, we mentioned that we would donate €1 per completed questionnaire to a charity organization for children with cancer in the city of Freiburg, Germany.

Participants were offered a summary of the results, and were asked, if their hospital would be interested to be contacted by the authors for a follow-up. In both cases they had to provide contact details.

### Measures

The questionnaire included the HLHO-10 items as conceptualized by Kowalski et al. [35] with a 7-point Likert scale from 1 (absolutely not) to 7 (to a very large extent). The further section included questions regarding structural and process characteristics of the hospital (e.g., size, type of legal entity) and the person that answers the questionnaire (type of director, years in the position at that hospital), as well as an essay question about the hospitals’ current activities to promote health literacy (Additional file 1 “Questionnaire”). Providing the hospital management with the opportunity to describe briefly institutional activities to improve organizational health literacy offered a glimpse inside what is happening in German hospitals with regard to OHL.

We undertook a pre-test of the survey methods and the questionnaire in *n* = 20 hospitals that were not included in the main survey. The pre-test did not indicate a need for change.

### Analysis

Numerical variables were assessed by descriptive analysis using mean, median, and percentages. The median score of the HLHO-10 questionnaire was calculated based on the scores of the 10 items. We focused the psychometric evaluation of the HLHO-10 on internal consistency (Cronbach’s alpha), i.e. the extent to which hospital managers provided consistent responses to the 10 items, and refrained from a complete assessment, since the original authors did a comprehensive psychometric analysis [35]. The association between different characteristics, such as number of years in post, hospital type (public, charitable, and for-profit) and the HLHO-10 score, was examined using statistical tests such as one-way analysis of variance (ANOVA) and the Kruskal–Wallis test. The outputs of the ANOVA test were subjected to a post-hoc analysis utilizing the Tukey test, which aimed to evaluate and discern significant differences among the variables. Followed by the Kruskal–Wallis test, the Dunn–Bonferroni test was used to illustrate the group differences.

To check for multiple answers from the same hospital, we analyzed the frequency of datasets with identical ‘(hospital) ownership’, ‘number of beds’, ‘quality management (system)’ but different ‘type of professional background resp. position’. Datasets that are identical in the first three variables but not on the fourth, might be from the same hospital.

All statistical analyses were performed using IBM^®^ SPSS^®^ 28.0 (IBM Corporation, Armonk, NY, USA) with Bonferroni correction and a significance level of 0.05.

To evaluate the essay question, we categorized the responses both deductively and inductively and then applied qualitative content analysis according to Kuckartz and Rädiker [38]. The category system comprised the above-mentioned ten attributes of the OHL concept according to Brach et al. [12], and was supplemented on the basis of the responses, e.g. with other health-related concepts such as occupational health management (for details see additional file 2 “category system”). All answers regarding current activities to enhance patient health literacy were processed by two researchers using MAXQDA 2022 (VERBI Software. Consult. Sozialforschung. GmbH, Berlin, Germany).

### Data protection and safety

All study participants were informed that participation in the study is voluntary and that there would be no linkage between their answers to the questionnaire and the respective hospital.

The voluntarily provided contact details were handled by an independent researcher not involved in the data analysis, and stored separately from the research data. This person also checked the free text answers for person- or institution-related information, and anonymized any such information.

### Reporting statements

The quantitative study is reported in accordance with the Strengthening the Reporting of Observational Studies in Epidemiology (STROBE) Statement [39], the Consolidated Criteria for Reporting Qualitative Research (COREQ, 32 items) were consulted for an essay question [40]. The completed STROBE statement and the COREQ statement are available on request from the corresponding author of this study (N.H.).

## Results

### Sample characteristics

In total, 371 of the 3,301 hospital managers completed the full questionnaire (11%). Of those, 116 (40%) were employed by charitable hospitals, 103 (36%) worked in public hospitals, and 71 (24%) were in for-profit hospitals. Table 1 summarizes the characteristics of the respondents. The majority of respondents are directors of nursing (49%) and have held their position for less than 5 years (33%). Managers of mid-sized hospitals, ranging from 200 to 499 beds, were most prominent amongst the respondents (46%). All of the hospitals had a quality management system in place, with more than three-quarters of hospitals adhering to the standards set by DIN EN ISO 9001 (78%).

Cases that are identical regarding the variables ‘number of beds’, ‘ownership’, and ‘(type of) quality management’, but not on the variable ‘position’, could be indicative for “duplicates”: more than one manager of a hospital answering the survey. Potential duplicates were found in less than 10% of the 371 cases. It seems reasonable to assume that we received one questionnaire per hospital and that the hospital manager who answered the survey represents the respective hospital.

**Table 1:** Characteristics of the sample (*n* = 371, due to missing values, the size of the total sample varies)

		*n*	%
		(*n* = 291)	100%
Position	Medical director	90	30.9
	Administrative director	42	14.4
	Director of Nursing	142	48.8
	Others	17	5.9
		(*n* = 290)	100%
Number of years in the position	Less than 5 years	95	32.8
	5 to 9 years	79	27.2
	10 to 15 years	44	15.2
	More than 15 years	72	24.8
		(*n* = 290)	100%
Ownership	Public	103	35.5
	Charitable	116	40.0
	For-profit	71	24.5
		(*n* = 289)	100%
Number of beds	Less than 100 beds	26	9.0
	100 to 199 beds	73	25.3
	200 to 499 beds	133	46.0
	500 and more beds	57	19.7
		(*n* = 253)	100%
Quality management	DIN EN ISO 9001	196	77.5
	DIN EN 15,224	5	2.0
	KTQ	34	13.4
	EFQM	3	1.2
	Others	15	5.9

### HLHO-10 results

The HLHO-10 shows satisfactory internal reliability, with Cronbach’s alpha at 0.88. Table 2 displays answers to the HLHO-10 survey items. On a scale from 1 (low OHL) to 7 (high OHL), the mean values range from 3.6 to 5.4. The average mean of the ten items is 4.6 (SD = 1.1).

The highest values are found for the sixth item “efforts made to ensure that patients can find their way at the hospital without any problems” (M = 5.2, SD = 1.3) and the ninth item “communicate openly and comprehensibly at the hospital to their patients in advance about the costs which they themselves have to pay for treatment” (M = 5.4, SD = 1.6). By contrast, the third item “involvement of patients in the development of health information” scores lowest (M = 3.6, SD = 1.6). Also, the individualized use of different media (No. 7) is seldom employed (M = 3.6, SD = 1.6). A small percentage of employees are trained in health literacy e.g., with communication training to support an informed decision (No. 10), (M = 3.7, SD = 1.7).

**Table 2:** Descriptive statistics on the HLHO-10 items

	To what extent…	Min	Max	Md	M	SD
1.	…is the management at your hospital explicitly dedicated to the subject of health literacy (e.g. mission statement, human resources planning)?	1	7	5.0	4.68	1.61
2.	…is the topic of health literacy considered in quality management measures at your hospital?	1	7	5.0	4.42	1.63
3.	…is health information at your hospital developed by involving patients?	1	7	4.0	3.59	1.63
4.	…is individualized health information used at your hospital (e.g. different languages, print sizes, braille)?	1	7	4.0	4.31	1.61
5.	…are there communication standards at your hospital which ensure that patients truly understand the necessary information (e.g. translators, allowing pauses for reflection, calling for further queries)?	1	7	5.0	4.53	1.57
6.	…are efforts made to ensure that patients can find their way at your hospital without any problems (e.g. direction signs, information staff)?	1	7	5.0	5.21	1.28
7.	…is information made available to different patients via different media at your hospital (e.g. three-dimensional models, DVDs, picture stories)?	1	7	4.0	3.64	1.61
8.	…is it ensured that the patients have truly understood everything, particularly in critical situations (e.g. medication, surgical consent), at your hospital?	1	7	5.0	5.10	1.35
9.	…do you communicate openly and comprehensibly at your hospital to your patients in advance about the costs which they themselves have to pay for treatment (e.g. out-of-pocket payments)?	1	7	6.0	5.35	1.55
10.	…are employees at your hospital trained on the topic of health literacy?	1	7	4.0	3.84	1.66

*Min* Minimum, *Max* Maximum, *Md* Median, *M* Mean, *SD* Standard deviation, *n* = 371

### Distinctions according to characteristics of the study

In a detailed analysis, we investigated differences in the response to HLHO-10 according to the characteristics of the sample.

#### Number of years in the position (Table 3)

 The number of years the participants have been in their position in the hospitals relates to the assessment of two HLHO-10 items. Firstly, the number of years in the current position relates to attribute No. 1 (“To what extent is the management of your hospital explicitly dedicated to the topic of health literacy (e.g., mission statement, personnel planning)?”), (F (3, 286) = 3.94, *p* = .009, ƞ^2^=0.04). In particular, the assessments of managers less than 5 years and between 5 and 9 years in their position differ from those who have been in the position for more than 15 years. Additionally, No. 7 (To what extent is information provided in your hospital via different media for different patients (e.g., three-dimensional models, DVDs, picture stories)? ), (F (3, 286) = 3.36, *p* = .019, ƞ^2^=0.034). Participants who have worked in the position for 5–9 years rated the variety of media lower than the group up to 5 years and the group more than 15 years in the current position.

**Table 3:** Distribution of HLHO-10 item scores by number of years in the position

HLHO-10 item	Less than 5 years	5 to 9 years	10 to 15 years	More than 15 years	F	p*
	Mean ± SD	Mean ± SD	Mean ± SD	Mean ± SD		
1. Leadership	4.54 ± 1.50	4.61 ± 1.62	4.66 ± 1.57	5.31 ± 1.53	3.942	0.009
2. Integration	4.32 ± 1.48	4.28 ± 1.88	4.57 ± 1.63	4.71 ± 1.51	1.189	0.314
3. Inclusion of served	3.42 ± 1.55	3.49 ± 1.89	3.66 ± 1.61	3.69 ± 1.56	0.464	0.707
4. Health literacy skills range	4.35 ± 1.53	4.27 ± 1.84	4.11 ± 1.48	4.40 ± 1.49	0.336	0.799
5. Communication standards	4.65 ± 1.51	4.46 ± 1.76	4.41 ± 1.40	4.49 ± 1.53	0.354	0.789
6. Provide access	5.33 ± 1.23	5.05 ± 1.39	5.30 ± 1.13	5.28 ± 1.25	0.784	0.504
7. Media variety	3.81 ± 1.43	3.15 ± 1.68	3.73 ± 1.60	3.85 ± 1.62	3.358	0.019
8. High-risk	5.03 ± 1.19	4.82 ± 1.56	5.39 ± 1.30	5.31 ± 1.23	2.499	0.060
9. Costs	5.28 ± 1.55	5.28 ± 1.64	5.43 ± 1.45	5.36 ± 1.45	0.131	0.942
10. Workforce	3.85 ± 1.60	3.54 ± 1.81	3.98 ± 1.58	4.18 ± 1.72	1.859	0.137

*SD *Standard deviation, *ANOVA - Post-hoc test with Bonferroni correction, sign. *p* < .05, *n* = 290

#### Ownership (Table 4)

The extent to which the management of a hospital is dedicated to health literacy (No. 1) differed significantly by hospital type (F (2, 287) = 3.61, *p* = .028, ƞ^2^=0.025). Managers of charitable hospitals stated higher values than managers of public or for-profit hospitals. Regarding the efforts of the hospitals to ensure that patients find their way around easily (No. 6), there was also a statistically significant difference according to hospital type (F (2, 287) = 3.22, *p* = .041, ƞ^2^=0.022). A distinction could be made between charitable and for-profit hospitals when compared with public hospitals, as the latter reported a lower level of easy orientation in their facility, based on participants’ feedback.

**Table 4:** Distribution of HLHO-10 item scores by hospital type

HLHO-10 item	Public	Charitable	For-Profit	F	p*
	Mean ± SD	Mean ± SD	Mean ± SD		
1. Leadership	4.60 ± 1.59	5.06 ± 1.45	4.51 ± 1.67	3.609	0.028
2. Integration	4.31 ± 1.60	4.65 ± 1.59	4.30 ± 1.72	1.544	0.215
3. Inclusion of served	3.52 ± 1.55	3.71 ± 1.68	3.35 ± 1.78	1.030	0.358
4. Health literacy skills range	4.26 ± 1.54	4.51 ± 1.48	4.03 ± 1.82	2.061	0.129
5. Communication standards	4.44 ± 1.52	4.6 ± 1.47	4.44 ± 1.79	0.672	0.512
6. Provide access	4.98 ± 1.35	5.39 ± 1.13	5.34 ± 1.31	3.220	0.041
7. Media variety	3.52 ± 1.45	3.60 ± 1.60	3.80 ± 1.76	0.658	0.518
8. High-risk	4.95 ± 1.38	5.11 ± 1.27	5.28 ± 1.37	1.301	0.274
9. Costs	5.25 ± 1.53	5.37 ± 1.49	5.35 ± 1.61	0.178	0.837
10. Workforce	3.81 ± 1.58	4.07 ± 1.76	3.62 ± 1.73	1.655	0.193

*SD *Standard deviation, *ANOVA - Post-hoc test with Bonferroni correction, sign. *p* < .05, *n* = 290

#### Quality management (Additional file 3 “Results tables”, Table S1)

The extent to which management addresses the issue of OHL (No. 1) shows in the first step significant differences by the type of quality management system (H (4) = 11.98; *p* = .018). The following post-hoc analysis revealed that no significant differences were found between the individual groups. Only differences in comparison to the category Other (quality management) between DIN EN ISO 9001 (*p* = .151) and DIN EN 15,224 (*p* = .244) were indicated.

No differences are found with regard to the characteristics of the OHL and the position in the hospital as well as the number of beds and thus the size of the hospital (Additional file 3 “results tables”, Tables S2 & S3).

### Free-text responses about hospitals’ practical OHL activities

120 of the 371 survey participants (32%) provided a total of *n* = 247 examples of health literacy activities, which we extracted as codes from the content analysis (Additional file 2 “category system”). Most of the activities were directed at effective communication (see Table 2, No. 5). In addition to information material (*n* = 17) and patient academies/information forums (*n* = 39), they also provide patient education (*n* = 19), e.g., on diabetes, to promote patients’ HL. Hospitals also make efforts to provide user-friendly materials (No. 7) by offering information not only verbally but also in written form or via (digital) video (*n* = 19). Furthermore, internal training courses (*n* = 12) are held for staff to train employees in health literacy-sensitive communication with patients (No.10). Further input was provided by hospital management on the topic of considering health literacy in quality management (No. 2). This is organized through the establishment of HL-promoting standards and questionnaires for patients (*n* = 10). In addition, the managers stated that they ensure easy access (No.6) by means of signposting systems and contact persons at reception (*n* = 8).

Few practical examples were found for the first attribute of leadership promoting OHL (*n* = 2) (No. 1), as well as for the attribute of active participation of the population in the development and evaluation of health information and services (*n* = 4) (No. 3), and the attribute of preventing high risk (*n* = 4) (No. 8). There were no practical examples for the attribute of taking measures to explain insurance coverage and costs (No. 9). In addition to the contents of HLHO-10 [35] and hence the 10 attributes according to Brach et al. [12], the concepts of workplace health management and workplace health promotion were mentioned as further topics (*n* = 9), e.g. offering an Employee Health and Fitness Day.

## Discussion

Organizational structures and processes in health care facilities have a significant influence on the promotion of health literacy [41]. To our knowledge, this is the first study to investigate the extent of OHL in German acute care hospitals nationwide. The self-assessment of management regarding the implementation of OHL shows areas in which OHL could be strengthened. Examples include the use of different media for communicating health information to patients or the training of employees on health literacy-related topics. While aspects of OHL are already embedded in inpatient healthcare within other frameworks (e.g. quality management systems, patient safety), more explicit attention to OHL is needed in the health care system to promote the initiatives of the hospitals [26, 42]. As with other change processes, the consolidation of the OHL approach requires a comprehensive organizational development strategy. This includes methods such as employee training and coaching on the topic of HL. It is evident that the management support and appropriate framework conditions are of significant importance in this context [25].

With regard to the degree of implementation of OHL in German hospitals, we can confirm the conclusion of previous research that there is a need for improvement in the area of OHL. When we compare our results with those of the HLHO-10 validation study [35], we find that in nine out of ten attributes of OHL German acute care hospitals in general score lower than certified breast cancer centres. This is also reflected in a higher mean score for Kowalski et al. [35], which was 4.9. As demonstrated in our own findings, Kowalski et al. [35] showed that patients are barely included in the development of health information. The result for the fourth question (Table 2, M = 4.31), concerning the use of individualized health information, is higher than that reported by Kowalski et al. (M(2015) = 3.57) [35]. Hence, we assume that in times of diversity-sensitive language, hospitals are also paying more attention to the topic [43]. In contrast with the findings of Kowalski et al. [35], our results illustrate substantial negative disparities in the domains of enforcing communication standards (see Table 2, No. 5) and obtaining patient consent in high-risk scenarios (No. 8). The poorer performance of the acute care hospitals in nine out of ten questions in the HLHO-10 comparison can be explained by the fact that Kowalski and colleagues [35] surveyed managers from specialized breast cancer centres. Furthermore, our study identified a significant correlation between HLHO-10 items and hospital ownership, as well as managers’ length of service. Nevertheless, there were no differences based on the type of position, suggesting that only one of the three hospital managers contacted responded, and that was the manager with the most comprehensive knowledge or a particular interest in health literacy, as illustrated by the large amount of responses in the free text answers.

Turning to international research on OHL using HLHO-10, it is evident that there are also differences in the degree of implementation of OHL worldwide [44]. For example, one study from Northern Texas with a sample of 74 key informants reported the same average rating of the items as our study: 4.6 [44]. However, in a study from Italy [45] among 405 healthcare managers the average HLHO-10 score was 5.4, considerable higher; the most notable differences were with regard to the provision of individualized health information (Table 2, No. 4) and to patient involvement in the development of patient-oriented materials (No. 3) [45]. Communication standards were more widely implemented and information was also provided to a greater extent through different media. Furthermore, in contrast to our study, the Italian study found a more significant difference between hospital types: 7 out of 10 items showed a significant difference, whereby private hospitals reported a higher level of OHL [45]. In line with our study, a smaller difference between the hospital types was found by a study with HLHO-10 from Turkey [46]. In this context, it should be mentioned that the results of international studies can vary considerably due to the different financing of different health care systems.

### Strengths and limitations

A strength of our study is the nationwide overview of the state of OHL in German hospitals, and the assessment of managers from different areas of responsibility. Another positive aspect is that the sample includes hospitals of different levels of care and of different sizes. While we included incentives during the recruitment of hospital managers [47, 48] and offered more flexible ways of participating in the study via online and offline channels [49, 50], potential additional support was restricted; for instance, hospital manager associations have very limited capacities, further reduced by the COVID-19 pandemic. This may also have impacted the response rate, as many staff were ill, exacerbating the staffing situation in German hospitals. Moreover, response rates in organizational surveys are known to be rather low and have decreased in recent decades [49]. Due to the framework of the project, a non-responder analysis was not feasible. In addition, the distribution of hospital types in our sample does not correspond to the German occurrence. Upon analysis of the data in comparison to the population of general hospitals in 2021 (*N* = 1,534), the distribution of hospital types in our sample does not reflect the situation in Germany. Specifically, for-profit hospitals constitute the majority of German hospitals (38%), followed by charitable hospitals (33%) and public hospitals (29%) [51]. However, in line with our data, the majority of hospitals operate within the range of 200 to 499 beds (32%) [51]. This is followed by hospitals with up to 99 beds (30%), up to 199 (22%), and finally exceeding 500 beds (16%) [51]. There are no reference values that can be directly compared with the prevalence of quality management systems. However, DIN ISO 9001:2015 is the most commonly used system, which corresponds with our study [52–54]. The reason for the low participation of private hospitals needs to be investigated.

## Conclusion

The key objective of this study was to investigate the state of implementation of OHL in German hospitals. The results indicate areas to be promoted in hospitals, including the participatory development of health information, its individual-centred use, and staff training on HL. Hospitals present excellent opportunities for reaching people, thus the promotion of OHL ought to comprise an essential aspect of the organizational culture and hold a fundamental position in the hospital’s daily functions. Due to the complexity of the healthcare system, hospitals must meet the complex needs, abilities, preferences, and medical requirements of their patients. The OHL assessment via HLHO-10 is a starting point, and therefore a status quo analysis. The findings of this study indicate that further implementation of the concept in hospital structures and processes is necessary. For example, strategies need to be developed to include patients in the development and evaluation of health information. Some guides and tools [34, 55] already exist to support the implementation of health literacy in organizations. Recent OHL research in Germany shows that quality management offers the structures and processes for the systematic implementation of OHL [56]. Furthermore, quality management can be employed to ensure and further develop the implementation of OHL standards. It is also important to note that most survey respondents exhibited a keen interest in advocating for OHL. The result of the initial question of HLHO-10 demonstrates a commitment to OHL and the answers to the essay question indicate a wide range of activities in the field of OHL. It is very important to support committed hospital managers and health professionals, and to sensitize other stakeholders from politics and healthcare facilities to the topic of OHL. Future research should also investigate OHL in other management levels of the hospitals and how they compare to results from patient surveys.

## Supplementary Information

Additional file 1. Questionnaire_Häberle_OHL. Questionnaire for the survey of hospital management. It contains the HLHO-10 items and other items.

Additional file 2. Category System OHL hospitals managers 2024.

Additional file 3. Results tables_Häberle_OHL. Results regarding the distribution of HLHO-10 item scores by quality management, position in the hospital and number of beds.

## Data Availability

The data presented in this study are available on reasonable request from the corresponding author.

## Abbreviations

HL: Health Literacy
HLHO-10: Health care organization ten item questionnaire
OHL: Organizational Health Literacy
