# Supplementary material for: Organizational health literacy in German hospitals: a cross-sectional survey among hospital managers
Source: BMC Health Serv Res. 2024 Oct 13;24:1227. doi: 10.1186/s12913-024-11649-x (PMC11475337; doi:10.1186/s12913-024-11649-x)
Supplement: Supplementary file 1 — Additional file 1. Questionnaire_Häberle_OHL. Questionnaire for the survey of hospital management. It contains the HLHO-10 items and other items. [file 12913_2024_11649_MOESM1_ESM.pdf]

Pädagogische Hochschule Freiburg
Université des Sciences de l'Education · University of Education

ChEG

MHH Medizinische Hochschule Hannover

Study "Enabling good healthcare decisions in hospitals" 2022

> **Health literacy** is the degree to which individuals are enabled by the educational, social and/or health care system to find, process and understand the health information relevant to making appropriate health-related decisions[1].

The following statements refer to measures that can support the health literacy of patients in hospital. When responding, please think about the location where you mainly work. The value 1 means "absolutely not", the value 7 means "to a very large extent". You can grade your assessment with the values in between.

1. To what extent…[2]	**Absolutely not** 1	**2**	**3**	**Partially** 4	**5**	**6**	**To a very large extent** 7
…is the management at your hospital explicitly dedicated to the subject of health literacy (e.g. mission statement, human resources planning)?	☐	☐	☐	☐	☐	☐	☐
…is the topic of health literacy considered in quality management measures at your hospital?	☐	☐	☐	☐	☐	☐	☐
…is health information at your hospital developed by involving patients?	☐	☐	☐	☐	☐	☐	☐
…is individualized health information used at your hospital (e.g. different languages, print sizes, braille)?	☐	☐	☐	☐	☐	☐	☐
…are there communication standards at your hospital which ensure that patients truly understand the necessary information (e.g. translators, allowing pauses for reflection, calling for further queries)?	☐	☐	☐	☐	☐	☐	☐
…are efforts made to ensure that patients can find their way at your hospital without any problems (e.g. direction signs, information staff)?	☐	☐	☐	☐	☐	☐	☐
…is information made available to different patients via different media at your hospital (e.g. three-dimensional models, DVDs, picture stories)?	☐	☐	☐	☐	☐	☐	☐

[1] Schaefer C, Bitzer EM, Dierks, M-L. Mehr Organisationale Gesundheitskompetenz in die Gesundheitsversorgung bringen! Ein Positionspapier des DNGK. 2019. https://dngk.de/gesundheitskompetenz/organisationale-gesundheitskompetenz-positionspapier-2019/. Accessed 5 Jul 2021.

[2] Kowalski C, Lee S-YD, Schmidt A, Wesselmann S, Wirtz MA, Pfaff H, Ernstmann N. The health literate health care organization 10 item questionnaire (HLHO-10): development and validation. BMC Health Serv Res. 2015;15:47. doi:10.1186/s12913-015-0707-5.

…is it ensured that the patients have truly understood everything, particularly in critical situations (e.g. medication, surgical consent), at your hospital?	☐	☐	☐	☐	☐	☐	☐
…do you communicate openly and comprehensibly at your hospital to your patients in advance about the costs which they themselves have to pay for treatment (e.g. out-of-pocket payments)?	☐	☐	☐	☐	☐	☐	☐
…are employees at your hospital trained on the topic of health literacy?	☐	☐	☐	☐	☐	☐	☐

Please introduce your own activity:

2. Is there anything that you have done until now to promote the health literacy of patients at the hospital where you work?
   If yes: please describe this activity (e.g. a project, a campaign) in keywords.

   ____

   ____

   ____

Information about you personally and the hospital where you work

Now we would like to find out more about you and your hospital and would be pleased if you could provide us further information.

3. In which area of the hospital do you work?	Medical director .... ☐<br>Administrative director .... ☐<br>Director of Nursing .... ☐<br>Other area, namely<br>____________________
4. How long (number of years) have you been working in this position?	Less than 5 years .... ☐<br>5 to 9 years .... ☐<br>10 to 15 years .... ☐<br>More than 15 years .... ☐
5. In which ownership is the hospital where you work located?	Public .... ☐<br>Charitable .... ☐<br>For-profit .... ☐
6. How many beds does the location where you work have?	Less than 100 beds .... ☐<br>100 to 199 beds .... ☐<br>200 to 499 beds .... ☐<br>500 and more beds .... ☐
7. Which quality management system does the location where you mainly operate work with?	DIN EN ISO 9001 .... ☐<br>DIN EN 15224 .... ☐<br>KTQ .... ☐<br>EFQM .... ☐<br>Others, namely<br>____________________
