# Supplementary material for: Organizational health literacy in German hospitals: a cross-sectional survey among hospital managers
Source: BMC Health Serv Res. 2024 Oct 13;24:1227. doi: 10.1186/s12913-024-11649-x (PMC11475337; doi:10.1186/s12913-024-11649-x)
Supplement: Supplementary file 2 — Additional file 2. Category System OHL hospitals managers 2024. [file 12913_2024_11649_MOESM2_ESM.pdf]

**Category system**
**Organizational health literacy in German hospitals: A cross-sectional survey among hospital managers**

| No. | Main category | Sub-category | Definition | Anchor quotations |
|---|---|---|---|---|
| 1 | HLO | | Based on: Brach et al. (2012) | |
| 1.1 | | Leadership promotes HL | The hospital manager describes how health literacy becomes an integral part of the mission statement, structures and processes. | *“Communication matrix and transparency in the mission statement of our clinics. "We for your health."* |
| 1.2 | | Plans, evaluates and improves | The hospital manager describes the actions taken to integrate health literacy into strategic planning, evaluation measures, patient safety and quality development. | *“Patient questionnaires, focus interviews with patients.”* |
| 1.3 | | Prepares the staff | The hospital manager describes what is being done to promote health literacy among staff and to achieve improvements. | *“Communication training and accompanying measures (e.g. creating space for discussions, explanations/information in other languages, visualisations).”* |
| 1.4 | | Practises participation | Hospital manager describes their activities to develop, implement and evaluate health information programmes with the involvement of target groups. | *“Patient participation in decision-making processes and cooperation with self-help groups.”* |
| 1.5 | | Meets the needs of the population | The hospital management describes its measures in terms of needs-orientated methods to promote the health literacy of those population groups that use the services of the facility | *“The Patient Information Centre is networked with numerous advice centres and self-help groups and organises many information events on health and illness topics as well as for young parents. The Patient Information Centre offers special advice on the topics of stopping smoking and health care proxy/patient decree/care decree. Advice at the Patient Information Centre is always free of charge.”* |
| 1.6 | | Communicates effectively | The hospital management outlines strategies to promote health literacy in interpersonal communication situations, or describes measures aimed at re-confirming correct understanding. | *„Cooperation with local newspapers on current topics, but also general medical topics. So-called evening consultation hours (information consultations, in person or via the web) on important health topics for patients and relatives/public.“* |
| 1.7 | | Ensures easy access | Hospital leadership describes easy access to health information and services, or how patients are supported to navigate the organisation. | *„Individual training of patients by specialist, or disease-related & Images on the doors.“* |

| | | | | |
|---|---|---|---|---|
| 1.8 | | Designs user-friendly materials | The hospital management describes measures for the development and use of printed, audiovisual and social media whose messages are easy to understand and follow. | *„Provision of extensive educational material, also in foreign languages."* |
| 1.9 | | Counteracts high risk | Hospital leadership describes health literacy interventions in high-risk settings, including gaps and interfaces in care and communication of medication information. | *„Accompanied discharge: care training for carers + patiens"* |
| 1.10 | | Explains insurance cover and costs | The hospital management describes measures to communicate which services are covered by insurance benefit catalogues and which services have to be paid for by the patients themselves. | No mentions |
| 2 | Health at work | | | |
| 2.1 | | Workplace health management | The hospital management describes measures for occupational health management. | *„Occupational health management including the health programme of individual health insurance companies. "* |
| 2.2 | | Workplace health promotion | The hospital management describes measures for workplace health promotion. | *„Various courses (e.g. Pilates, yoga, etc.) at our training centre."* |
