# Supplementary material for: Organizational health literacy in German hospitals: a cross-sectional survey among hospital managers
Source: BMC Health Serv Res. 2024 Oct 13;24:1227. doi: 10.1186/s12913-024-11649-x (PMC11475337; doi:10.1186/s12913-024-11649-x)
Supplement: Supplementary file 3 — Additional file 3. Results tables_Häberle_OHL. Results regarding the distribution of HLHO-10 item scores by quality management, position in the hospital and number of beds. [file 12913_2024_11649_MOESM3_ESM.pdf]

Table S1 Results of the analysis of HLHO-10 item scores by quality management

HLHO-10 item	H-value	df	p*
1. Leadership	11.979	4	.018
2. Integration	7.681	4	.104
3. Inclusion of served	3.018	4	.555
4. Health literacy skills range	6.159	4	.188
5. Communication standards	3.266	4	.514
6. Provide access	.0955	4	.917
7. Media variety	2.999	4	.558
8. High-risk	3.232	4	.520
9. Costs	2.163	4	.706
10. Workforce	8.812	4	.066

*Kruskal-Wallis test, n=253

Table S2 Distribution of HLHO-10 item scores by position in the hospital

HLHO-10 item	Medical director Mean ± SD	Administrative director Mean ± SD	Director of Nursing Mean ± SD	Others Mean ± SD	F	p*
1. Leadership	4.77 ± 1.48	4.71 ± 1.54	4.76 ± 1.65	4.88 ± 1.54	.046	.987
2. Integration	4.57 ± 1.64	4.50 ± 1.58	4.32 ± 1.65	4.82 ± 1.55	.781	.505
3. Inclusion of served	3.86 ± 1.77	3.43 ± 1.66	3.37 ± 1.60	4.00 ± 1.54	2.088	.102
4. Health literacy skills range	4.67 ± 1.53	4.12 ± 1.67	4.16 ± 1.59	4.29 ± 1.61	2.138	.096
5. Communication standards	4.80 ± 1.60	4.38 ± 1.56	4.46 ± 1.60	4.18 ± 1.24	1.374	.251
6. Provide access	5.37 ± 1.25	5.26 ± 1.17	5.13 ± 1.32	5.41 ± 1.12	0.739	.529
7. Media variety	3.81 ± 1.56	3.69 ± 1.68	3.54 ± 1.56	3.47 ± 1.91	.624	.600
8. High-risk	5.19 ± 1.30	5.45 ± 1.29	4.94 ± 1.36	5.12 ± 1.36	1.772	.153
9. Costs	5.34 ± 1.64	5.69 ± 1.18	5.17 ± 1.55	5.71 ± 1.36	1.663	.175
10. Workforce	3.82 ± 1.66	4.05 ± 1.75	3.80 ± 1.73	4.41 ± 1.37	.829	.479

SD= Standard deviation, *ANOVA, n=291

Table S3 Distribution of HLHO-10 item scores by number of beds

HLHO-10 item	Less than 100 beds Mean ± SD	100 to 200 beds Mean ± SD	200 to 499 beds Mean ± SD	500 and more beds Mean ± SD	F	p*
1. Leadership	4.77 ± 1.42	4.81 ± 1.57	4.75 ± 1.61	4.70 ± 1.60	.050	.985
2. Integration	4.62 ± 1.55	4.49 ± 1.65	4.42 ± 1.63	4.35 ± 1.64	.187	.905
3. Inclusion of served	3.81 ± 1.89	3.47 ± 1.75	3.54 ± 1.64	3.63± 1.58	.305	.822
4. Health literacy skills range	3.81 ± 1.744	4.14 ± 1.64	4.44 ± 1.60	4.47 ± 1.47	1.62	.184
5. Communication standards	4.73 ± 1.78	4.47 ± 1.60	4.48 ± 1.62	4.61 ± 1.37	.275	.844
6. Provide access	5.46 ± 1.17	5.40 ± 1.11	5.14 ± 1.33	5.12 ± 1.34	1.057	.368
7. Media variety	3.46 ± 1.45	3.49 ± 1.67	3.62 ± 1.59	3.86 ± 1.59	.665	.574
8. High-risk	5.04 ± 1.22	5.18 ± 1.31	5.05 ± 1.39	5.12 ± 1.32	.178	.911
9. Costs	5.62 ± 1.33	5.11 ± 1.75	5.40 ± 1.39	5.28 ± 1.62	.913	.435
10. Workforce	3.35 ± 1.65	3.85 ± 1.78	3.90 ± 1.74	4.02 ± 1.70	.988	.399

SD= Standard deviation, *ANOVA, n=289
